# Supplementary material for: Migraine eye: correlation between migraine and the retina
Source: PeerJ. 2024 May 27;12:e17454. doi: 10.7717/peerj.17454 (PMC11138520; doi:10.7717/peerj.17454)
Supplement: Supplemental Information 3 — The data indicate the thickness of retinal sector in migraine patient with unilateral headache to compare between headache-side eye, non-headache-side eye and control eye. [file peerj-12-17454-s003.docx]

**Table S1**

**Caption**: Comparison of thicknesses of each retinal layer of controls, headache-side eyes, and non-headache-side eyes of migraine patients.

| **Layers and Sectors** | | | **Mean Difference (µm)** | **Standard Error** | **p value** | **95% Confidence Interval** | |
| --- | --- | --- | --- | --- | --- | --- | --- |
|  |  |  |  |  |  | **Lower Bound** | **Upper Bound** |
| pRNFL G | Control | Headache-side eye | -0.72 | 2.91 | 0.804 | -6.514 | 5.067 |
|  | Control | Non-headache-side eye | -0.17 | 2.91 | 0.953 | -5.964 | 5.617 |
|  | Headache-side eye | Non-headache-side eye | 0.55 | 3.33 | 0.869 | -6.078 | 7.178 |
| pRNFL T | Control | Headache-side eye | -2.53 | 3.85 | 0.514 | -10.206 | 5.148 |
|  | Control | Non-headache-side eye | 1.27 | 3.85 | 0.742 | -6.406 | 8.948 |
|  | Headache-side eye | Non-headache-side eye | 3.80 | 4.41 | 0.392 | -4.988 | 12.588 |
| pRNFL TS | Control | Headache-side eye | 1.31 | 4.82 | 0.786 | -8.294 | 10.920 |
|  | Control | Non-headache-side eye | 0.76 | 4.82 | 0.875 | -8.844 | 10.370 |
|  | Headache-side eye | Non-headache-side eye | -0.55 | 5.52 | 0.921 | -11.547 | 10.447 |
| pRNFL NS | Control | Headache-side eye | -0.58 | 4.79 | 0.903 | -10.127 | 8.959 |
|  | Control | Non-headache-side eye | -3.88 | 4.79 | 0.420 | -13.427 | 5.659 |
|  | Headache-side eye | Non-headache-side eye | -3.30 | 5.48 | 0.549 | -14.224 | 7.624 |
| pRNFL N | Control | Headache-side eye | 1.03 | 3.68 | 0.780 | -6.299 | 8.357 |
|  | Control | Non-headache-side eye | -1.12 | 3.68 | 0.761 | -8.449 | 6.207 |
|  | Headache-side eye | Non-headache-side eye | -2.15 | 4.21 | 0.611 | -10.538 | 6.238 |
| pRNFL IN | Control | Headache-side eye | 7.96 | 5.48 | 0.151 | -2.957 | 18.868 |
|  | Control | Non-headache-side eye | 8.26 | 5.48 | 0.136 | -2.657 | 19.168 |
|  | Headache-side eye | Non-headache-side eye | 0.30 | 6.27 | 0.962 | -12.192 | 12.792 |
| pRNFL TI | Control | Headache-side eye | -1.31 | 4.94 | 0.792 | -11.138 | 8.527 |
|  | Control | Non-headache-side eye | 4.14 | 4.94 | 0.404 | -5.688 | 13.977 |
|  | Headache-side eye | Non-headache-side eye | 5.45 | 5.65 | 0.338 | -5.805 | 16.705 |
| GCL cen | Control | Headache-side eye | 1.22 | 1.09 | 0.264 | -.944 | 3.391 |
|  | Control | Non-headache-side eye | 0.18 | 1.07 | 0.864 | -1.947 | 2.315 |
|  | Headache-side eye | Non-headache-side eye | -1.04 | 1.24 | 0.405 | -3.511 | 1.432 |
| GCL Tinn | Control | Headache-side eye | -0.74 | 1.30 | 0.574 | -3.335 | 1.861 |
|  | Control | Non-headache-side eye | 0.61 | 1.28 | 0.634 | -1.941 | 3.167 |
|  | Headache-side eye | Non-headache-side eye | 1.35 | 1.49 | 0.367 | -1.612 | 4.312 |
| GCL TSinn | Control | Headache-side eye | 0.11 | 1.05 | 0.920 | -1.979 | 2.190 |
|  | Control | Non-headache-side eye | -0.08 | 1.03 | 0.937 | -2.131 | 1.968 |
|  | Headache-side eye | Non-headache-side eye | -0.19 | 1.19 | 0.876 | -2.564 | 2.190 |
| GCL Ninn | Control | Headache-side eye | 0.51 | 1.21 | 0.672 | -1.890 | 2.916 |
|  | Control | Non-headache-side eye | -0.78 | 1.19 | 0.512 | -3.144 | 1.581 |
|  | Headache-side eye | Non-headache-side eye | -1.29 | 1.38 | 0.349 | -4.035 | 1.445 |
| GCL Iinn | Control | Headache-side eye | -0.92 | 1.19 | 0.441 | -3.288 | 1.446 |
|  | Control | Non-headache-side eye | 0.11 | 1.17 | 0.927 | -2.220 | 2.435 |
|  | Headache-side eye | Non-headache-side eye | 1.03 | 1.35 | 0.450 | -1.670 | 3.728 |
| GCL Tout | Control | Headache-side eye | 2.1447^*^ | 1.04 | 0.042* | .077 | 4.212 |
|  | Control | Non-headache-side eye | 1.46 | 1.02 | 0.158 | -.578 | 3.488 |
|  | Headache-side eye | Non-headache-side eye | -0.69 | 1.18 | 0.562 | -3.047 | 1.668 |
| GCL Sout | Control | Headache-side eye | -0.14 | 0.95 | 0.879 | -2.037 | 1.748 |
|  | Control | Non-headache-side eye | 0.41 | 0.93 | 0.666 | -1.455 | 2.266 |
|  | Headache-side eye | Non-headache-side eye | 0.55 | 1.08 | 0.613 | -1.608 | 2.708 |
| GCL Nout | Control | Headache-side eye | 0.61 | 0.96 | 0.531 | -1.309 | 2.519 |
|  | Control | Non-headache-side eye | 0.81 | 0.94 | 0.394 | -1.071 | 2.692 |
|  | Headache-side eye | Non-headache-side eye | 0.21 | 1.10 | 0.852 | -1.977 | 2.388 |
| GCL Iout | Control | Headache-side eye | 1.30 | 0.85 | 0.131 | -.397 | 3.003 |
|  | Control | Non-headache-side eye | 1.19 | 0.84 | 0.160 | -.479 | 2.864 |
|  | Headache-side eye | Non-headache-side eye | -0.11 | 0.97 | 0.910 | -2.049 | 1.828 |
| mRNFL cen | Control | Headache-side eye | 1.25 | 1.46 | 0.394 | -1.657 | 4.157 |
|  | Control | Non-headache-side eye | 1.27 | 1.43 | 0.380 | -1.592 | 4.124 |
|  | Headache-side eye | Non-headache-side eye | 0.02 | 1.66 | 0.992 | -3.299 | 3.330 |
| mRNFL Tinn | Control | Headache-side eye | 0.24 | 0.29 | 0.419 | -.343 | .817 |
|  | Control | Non-headache-side eye | 0.16 | 0.29 | 0.589 | -.415 | .726 |
|  | Headache-side eye | Non-headache-side eye | -0.08 | 0.33 | 0.807 | -.743 | .580 |
| mRNFL TSinn | Control | Headache-side eye | 1.58 | 1.00 | 0.117 | -.405 | 3.562 |
|  | Control | Non-headache-side eye | 1.07 | 0.98 | 0.277 | -.879 | 3.021 |
|  | Headache-side eye | Non-headache-side eye | -0.51 | 1.13 | 0.656 | -2.769 | 1.754 |
| mRNFL Ninn | Control | Headache-side eye | -0.20 | 0.62 | 0.750 | -1.429 | 1.034 |
|  | Control | Non-headache-side eye | 0.31 | 0.61 | 0.611 | -.901 | 1.522 |
|  | Headache-side eye | Non-headache-side eye | 0.51 | 0.70 | 0.473 | -.897 | 1.912 |
| mRNFL Iinn | Control | Headache-side eye | -0.79 | 0.77 | 0.308 | -2.322 | .743 |
|  | Control | Non-headache-side eye | -0.63 | 0.76 | 0.408 | -2.136 | .878 |
|  | Headache-side eye | Non-headache-side eye | 0.16 | 0.88 | 0.855 | -1.587 | 1.908 |
| mRNFL Tout | Control | Headache-side eye | 0.41 | 0.55 | 0.458 | -.681 | 1.497 |
|  | Control | Non-headache-side eye | 0.27 | 0.54 | 0.622 | -.805 | 1.336 |
|  | Headache-side eye | Non-headache-side eye | -0.14 | 0.62 | 0.820 | -1.383 | 1.099 |
| mRNFL Sout | Control | Headache-side eye | 3.4737^*^ | 1.64 | 0.038* | .205 | 6.742 |
|  | Control | Non-headache-side eye | 2.95 | 1.61 | 0.071 | -.264 | 6.164 |
|  | Headache-side eye | Non-headache-side eye | -0.52 | 1.87 | 0.780 | -4.250 | 3.203 |
| mRNFL Nout | Control | Headache-side eye | 0.50 | 1.82 | 0.785 | -3.131 | 4.131 |
|  | Control | Non-headache-side eye | 0.87 | 1.79 | 0.630 | -2.704 | 4.436 |
|  | Headache-side eye | Non-headache-side eye | 0.37 | 2.08 | 0.861 | -3.774 | 4.506 |
| mRNFL Iout | Control | Headache-side eye | 0.29 | 1.50 | 0.848 | -2.706 | 3.285 |
|  | Control | Non-headache-side eye | 0.06 | 1.48 | 0.970 | -2.890 | 3.000 |
|  | Headache-side eye | Non-headache-side eye | -0.23 | 1.71 | 0.892 | -3.649 | 3.181 |
| INL cen | Control | Headache-side eye | 0.72 | 1.24 | 0.561 | -1.743 | 3.191 |
|  | Control | Non-headache-side eye | 0.42 | 1.22 | 0.734 | -2.010 | 2.841 |
|  | Headache-side eye | Non-headache-side eye | -0.31 | 1.41 | 0.828 | -3.121 | 2.505 |
| INL Tinn | Control | Headache-side eye | -0.20 | 1.00 | 0.845 | -2.197 | 1.803 |
|  | Control | Non-headache-side eye | 0.83 | 0.99 | 0.401 | -1.132 | 2.801 |
|  | Headache-side eye | Non-headache-side eye | 1.03 | 1.14 | 0.370 | -1.249 | 3.312 |
| INL TSinn | Control | Headache-side eye | -0.34 | 0.98 | 0.728 | -2.293 | 1.609 |
|  | Control | Non-headache-side eye | 0.23 | 0.96 | 0.808 | -1.684 | 2.153 |
|  | Headache-side eye | Non-headache-side eye | 0.58 | 1.12 | 0.607 | -1.648 | 2.801 |
| INL Ninn | Control | Headache-side eye | 0.76 | 0.96 | 0.429 | -1.148 | 2.674 |
|  | Control | Non-headache-side eye | -0.18 | 0.94 | 0.846 | -2.063 | 1.695 |
|  | Headache-side eye | Non-headache-side eye | -0.95 | 1.09 | 0.389 | -3.126 | 1.231 |
| INL Iinn | Control | Headache-side eye | 0.76 | 0.93 | 0.415 | -1.092 | 2.618 |
|  | Control | Non-headache-side eye | 0.05 | 0.92 | 0.957 | -1.774 | 1.874 |
|  | Headache-side eye | Non-headache-side eye | -0.71 | 1.06 | 0.504 | -2.828 | 1.402 |
| INL Tout | Control | Headache-side eye | 0.46 | 0.62 | 0.462 | -.780 | 1.701 |
|  | Control | Non-headache-side eye | 0.40 | 0.61 | 0.516 | -.820 | 1.620 |
|  | Headache-side eye | Non-headache-side eye | -0.06 | 0.71 | 0.932 | -1.475 | 1.354 |
| INL Sout | Control | Headache-side eye | -0.87 | 0.70 | 0.218 | -2.261 | .524 |
|  | Control | Non-headache-side eye | -1.24 | 0.69 | 0.075 | -2.611 | .127 |
|  | Headache-side eye | Non-headache-side eye | -0.37 | 0.80 | 0.640 | -1.961 | 1.214 |
| INL Nout | Control | Headache-side eye | 0.55 | 0.79 | 0.486 | -1.020 | 2.125 |
|  | Control | Non-headache-side eye | 0.69 | 0.78 | 0.374 | -.851 | 2.241 |
|  | Headache-side eye | Non-headache-side eye | 0.14 | 0.90 | 0.875 | -1.651 | 1.935 |
| INL Iout | Control | Headache-side eye | 1.12 | 0.67 | 0.100 | -.220 | 2.456 |
|  | Control | Non-headache-side eye | 0.56 | 0.66 | 0.401 | -.758 | 1.873 |
|  | Headache-side eye | Non-headache-side eye | -0.56 | 0.77 | 0.466 | -2.086 | .965 |

* = significant

Cen = center

Inn = inner

Out = outer

G = global

T = temporal

TS = temporal-superior

NS = nasal-superior

N = nasal

NI = nasal-inferior

TI = temporal-inferior
