# Supplementary material for: Migraine eye: correlation between migraine and the retina
Source: PeerJ. 2024 May 27;12:e17454. doi: 10.7717/peerj.17454 (PMC11138520; doi:10.7717/peerj.17454)
Supplement: Supplemental Information 4 — 4 tables that showed the statistical result when combine nasal and lateral side of the retina respect to the optic pathway, compare between headache-side eye (innervating), non-headache-side eye (non-innervating) and control. [file peerj-12-17454-s004.docx]

**Table S2**

**Caption:** Optic pathway of migraine patients compared to controls

| **Group Statistics** | | | | | | | |
| --- | --- | --- | --- | --- | --- | --- | --- |
| **Paramaters** | | | **Statistic** | **Bootstrapa** | | | |
|  |  |  |  | **Bias** | **Std. Error** | **95% Confidence Interval** | |
|  |  |  |  |  |  | **Lower** | **Upper** |
| Innervating pRNFL | Migraine | N | 19 |  |  |  |  |
|  |  | Mean | 102.458 | .004 | 2.065 | 98.485 | 106.581 |
|  |  | Std. Deviation | 9.2671 | -.3398 | 1.2388 | 6.4761 | 11.2820 |
|  |  | Std. Error Mean | 2.1260 |  |  |  |  |
|  | Control | N | 38 |  |  |  |  |
|  |  | Mean | 102.978 | -.013 | 1.505 | 100.076 | 105.930 |
|  |  | Std. Deviation | 9.5577 | -.1818 | 1.2168 | 7.1444 | 11.9272 |
|  |  | Std. Error Mean | 1.5505 |  |  |  |  |
| Non-innervating pRNFL | Migraine | N | 19 |  |  |  |  |
|  |  | Mean | 100.797 | .024 | 1.881 | 97.209 | 104.651 |
|  |  | Std. Deviation | 8.1139 | -.3102 | 1.2607 | 4.9814 | 10.2090 |
|  |  | Std. Error Mean | 1.8615 |  |  |  |  |
|  | Control | N | 38 |  |  |  |  |
|  |  | Mean | 102.978 | -.013 | 1.505 | 100.076 | 105.930 |
|  |  | Std. Deviation | 9.5577 | -.1818 | 1.2168 | 7.1444 | 11.9272 |
|  |  | Std. Error Mean | 1.5505 |  |  |  |  |
| Innervating mRNFL | Migraine | N | 19 |  |  |  |  |
|  |  | Mean | 35.856 | -.053 | 1.859 | 32.132 | 39.423 |
|  |  | Std. Deviation | 8.1894 | -.2664 | .8402 | 6.0785 | 9.5136 |
|  |  | Std. Error Mean | 1.8788 |  |  |  |  |
|  | Control | N | 38 |  |  |  |  |
|  |  | Mean | 34.777 | -.033 | .670 | 33.371 | 36.130 |
|  |  | Std. Deviation | 4.2780 | -.1291 | .8038 | 2.7063 | 5.7690 |
|  |  | Std. Error Mean | .6940 |  |  |  |  |
| Non-innervating mRNFL | Migraine | N | 19 |  |  |  |  |
|  |  | Mean | 33.115 | -.003 | 1.068 | 31.087 | 35.239 |
|  |  | Std. Deviation | 4.5649 | -.1871 | .7946 | 2.7943 | 5.8466 |
|  |  | Std. Error Mean | 1.0473 |  |  |  |  |
|  | Control | N | 38 |  |  |  |  |
|  |  | Mean | 34.777 | -.033 | .670 | 33.371 | 36.130 |
|  |  | Std. Deviation | 4.2780 | -.1291 | .8038 | 2.7063 | 5.7690 |
|  |  | Std. Error Mean | .6940 |  |  |  |  |
| Innervating GCL | Migraine | N | 19 |  |  |  |  |
|  |  | Mean | 40.252 | .022 | .599 | 39.151 | 41.479 |
|  |  | Std. Deviation | 2.6494 | -.1011 | .4184 | 1.7075 | 3.3783 |
|  |  | Std. Error Mean | .6078 |  |  |  |  |
|  | Control | N | 38 |  |  |  |  |
|  |  | Mean | 41.183 | -.026 | .461 | 40.300 | 42.084 |
|  |  | Std. Deviation | 2.9766 | -.0680 | .2914 | 2.3092 | 3.4327 |
|  |  | Std. Error Mean | .4829 |  |  |  |  |
| Non-innervating GCL | Migraine | N | 19 |  |  |  |  |
|  |  | Mean | 40.311 | .019 | .635 | 38.943 | 41.435 |
|  |  | Std. Deviation | 2.8559 | -.2432 | .8587 | 1.2734 | 4.2718 |
|  |  | Std. Error Mean | .6552 |  |  |  |  |
|  | Control | N | 38 |  |  |  |  |
|  |  | Mean | 41.183 | -.026 | .461 | 40.300 | 42.084 |
|  |  | Std. Deviation | 2.9766 | -.0680 | .2914 | 2.3092 | 3.4327 |
|  |  | Std. Error Mean | .4829 |  |  |  |  |
| Innervating INL | Migraine | N | 19 |  |  |  |  |
|  |  | Mean | 35.241 | .024 | .577 | 34.199 | 36.427 |
|  |  | Std. Deviation | 2.5103 | -.1025 | .3748 | 1.6908 | 3.1403 |
|  |  | Std. Error Mean | .5759 |  |  |  |  |
|  | Control | N | 38 |  |  |  |  |
|  |  | Mean | 35.692 | -.006 | .364 | 34.957 | 36.396 |
|  |  | Std. Deviation | 2.2329 | -.0476 | .1875 | 1.8004 | 2.5335 |
|  |  | Std. Error Mean | .3622 |  |  |  |  |
| No-innervating INL | Migraine | N | 19 |  |  |  |  |
|  |  | Mean | 35.171 | .012 | .456 | 34.304 | 36.164 |
|  |  | Std. Deviation | 2.0064 | -.0829 | .2790 | 1.3545 | 2.4872 |
|  |  | Std. Error Mean | .4603 |  |  |  |  |
|  | Control | N | 38 |  |  |  |  |
|  |  | Mean | 35.692 | -.006 | .364 | 34.957 | 36.396 |
|  |  | Std. Deviation | 2.2329 | -.0476 | .1875 | 1.8004 | 2.5335 |
|  |  | Std. Error Mean | .3622 |  |  |  |  |
| a. Unless otherwise noted, bootstrap results are based on 1000 bootstrap samples | | | | | | | |

**Table S3**

**Caption:** Optic pathway of migraine patients compared to controls

| **Independent Samples Test** | | | | | | | | | | |
| --- | --- | --- | --- | --- | --- | --- | --- | --- | --- | --- |
| **Parameters** | | **Levene's Test for Equality of Variances** | | **t-test for Equality of Means** | | | | | | |
|  |  | **F** | **p-value** | **t** | **df** | **p-value (2-tailed)** | **Mean Difference** | **Standard Error Difference** | **95% Confidence Interval** | |
|  |  |  |  |  |  |  |  |  | **Lower** | **Upper** |
| Innervating pRNFL | Equal variances assumed | .002 | .965 | -.196 | 55 | .845 | -.5208 | 2.6590 | -5.8497 | 4.8080 |
|  | Equal variances not assumed |  |  | -.198 | 37.129 | .844 | -.5208 | 2.6313 | -5.8518 | 4.8101 |
| Non-innervating pRNFL | Equal variances assumed | .876 | .353 | -.852 | 55 | .398 | -2.1817 | 2.5598 | -7.3116 | 2.9483 |
|  | Equal variances not assumed |  |  | -.901 | 41.842 | .373 | -2.1817 | 2.4226 | -7.0712 | 2.7079 |
| Innervating mRNFL | Equal variances assumed | 19.370 | .000 | .656 | 55 | .514 | 1.0791 | 1.6446 | -2.2168 | 4.3750 |
|  | Equal variances not assumed |  |  | .539 | 23.038 | .595 | 1.0791 | 2.0028 | -3.0637 | 5.2220 |
| Non-innervating mRNFL | Equal variances assumed | .439 | .510 | -1.353 | 55 | .182 | -1.6622 | 1.2290 | -4.1252 | .8007 |
|  | Equal variances not assumed |  |  | -1.323 | 34.083 | .195 | -1.6622 | 1.2563 | -4.2152 | .8907 |
| Innervating GCL | Equal variances assumed | .271 | .605 | -1.154 | 55 | .254 | -.9314 | .8074 | -2.5495 | .6867 |
|  | Equal variances not assumed |  |  | -1.200 | 40.117 | .237 | -.9314 | .7763 | -2.5001 | .6374 |
| Non-innervating GCL | Equal variances assumed | 1.038 | .313 | -1.057 | 55 | .295 | -.8727 | .8254 | -2.5269 | .7814 |
|  | Equal variances not assumed |  |  | -1.072 | 37.484 | .290 | -.8727 | .8139 | -2.5211 | .7757 |
| Innervating INL | Equal variances assumed | .358 | .552 | -.688 | 55 | .494 | -.4502 | .6539 | -1.7607 | .8603 |
|  | Equal variances not assumed |  |  | -.662 | 32.578 | .513 | -.4502 | .6803 | -1.8350 | .9347 |
| Non-innervating INL | Equal variances assumed | .413 | .523 | -.858 | 55 | .395 | -.5209 | .6073 | -1.7379 | .6962 |
|  | Equal variances not assumed |  |  | -.889 | 39.775 | .379 | -.5209 | .5857 | -1.7049 | .6632 |

**Table S4**

**Caption:** Optic pathway comparison between eyes of migraine patient

| **Paired Samples Statistics** | | | | | | | |
| --- | --- | --- | --- | --- | --- | --- | --- |
| **Parameters** | | | **Statistic** | **Bootstrapa** | | | |
|  |  |  |  | **Bias** | **Std. Error** | **95% Confidence Interval** | |
|  |  |  |  |  |  | **Lower** | **Upper** |
| Pair 1 | Innervating pRNFL | Mean | 102.458 | -.005 | 2.111 | 98.176 | 106.798 |
|  |  | N | 19 |  |  |  |  |
|  |  | Std. Deviation | 9.2671 | -.3241 | 1.2411 | 6.6954 | 11.6284 |
|  |  | Std. Error Mean | 2.1260 |  |  |  |  |
|  | Non-innervating pRNFL | Mean | 100.797 | -.002 | 1.824 | 97.155 | 104.595 |
|  |  | N | 19 |  |  |  |  |
|  |  | Std. Deviation | 8.1139 | -.3358 | 1.2389 | 5.3005 | 10.2281 |
|  |  | Std. Error Mean | 1.8615 |  |  |  |  |
| Pair 2 | Innervating mRNFL | Mean | 35.856 | -.092 | 1.874 | 31.936 | 39.483 |
|  |  | N | 19 |  |  |  |  |
|  |  | Std. Deviation | 8.1894 | -.3006 | .8489 | 6.0851 | 9.4353 |
|  |  | Std. Error Mean | 1.8788 |  |  |  |  |
|  | Non-innervating mRNFL | Mean | 33.115 | .034 | 1.090 | 31.131 | 35.481 |
|  |  | N | 19 |  |  |  |  |
|  |  | Std. Deviation | 4.5649 | -.1657 | .7946 | 2.7947 | 5.9386 |
|  |  | Std. Error Mean | 1.0473 |  |  |  |  |
| Pair 3 | Innervating GCL | Mean | 40.252 | -.025 | .598 | 39.122 | 41.420 |
|  |  | N | 19 |  |  |  |  |
|  |  | Std. Deviation | 2.6494 | -.1356 | .4027 | 1.7494 | 3.2815 |
|  |  | Std. Error Mean | .6078 |  |  |  |  |
|  | Non-innervating GCL | Mean | 40.311 | -.008 | .658 | 38.816 | 41.428 |
|  |  | N | 19 |  |  |  |  |
|  |  | Std. Deviation | 2.8559 | -.2314 | .8801 | 1.2785 | 4.3371 |
|  |  | Std. Error Mean | .6552 |  |  |  |  |
| Pair 4 | Innervating INL | Mean | 35.241 | -.012 | .559 | 34.066 | 36.270 |
|  |  | N | 19 |  |  |  |  |
|  |  | Std. Deviation | 2.5103 | -.1068 | .3610 | 1.7176 | 3.0989 |
|  |  | Std. Error Mean | .5759 |  |  |  |  |
|  | Non-innervating INL | Mean | 35.171 | -.001 | .448 | 34.262 | 36.018 |
|  |  | N | 19 |  |  |  |  |
|  |  | Std. Deviation | 2.0064 | -.0804 | .2747 | 1.3566 | 2.4490 |
|  |  | Std. Error Mean | .4603 |  |  |  |  |
| a. Unless otherwise noted, bootstrap results are based on 1000 bootstrap samples | | | | | | | |

**Table S5**

**Caption:** Optic pathway comparison between eyes of migraine patient

| **Paired Samples Test** | | | | | | | | | |
| --- | --- | --- | --- | --- | --- | --- | --- | --- | --- |
| **Parameters** | | **Paired Differences** | | | | | **t** | **df** | **p-value (2-tailed)** |
|  |  | **Mean** | **Standard Deviation** | **Standard Error Mean** | **95% Confidence Interval** | |  |  |  |
|  |  |  |  |  | **Lower** | **Upper** |  |  |  |
| Pair 1 | Innervating pRNFL - Non-innervating pRNFL | 1.6608 | 5.7455 | 1.3181 | -1.1084 | 4.4301 | 1.260 | 18 | .224 |
| Pair 2 | Innvervating mRNFL - Non-innervating mRNFL | 2.7414 | 10.6588 | 2.4453 | -2.3960 | 7.8787 | 1.121 | 18 | .277 |
| Pair 3 | Innervating GCL - Non-innervating GCL | -.0586 | 1.8565 | .4259 | -.9535 | .8362 | -.138 | 18 | .892 |
| Pair 4 | Innervating INL - Non-innervating INL | .0707 | 1.1834 | .2715 | -.4997 | .6411 | .260 | 18 | .798 |
