# Supplementary material for: Migraine eye: correlation between migraine and the retina
Source: PeerJ. 2024 May 27;12:e17454. doi: 10.7717/peerj.17454 (PMC11138520; doi:10.7717/peerj.17454)
Supplement: Supplemental Information 5 — This questionnaire was used to evaluate severity of migraine headache. [file peerj-12-17454-s005.docx]

**Thai version MIDAS Questionnaire**

เอกสารแนบหมายเลข 1


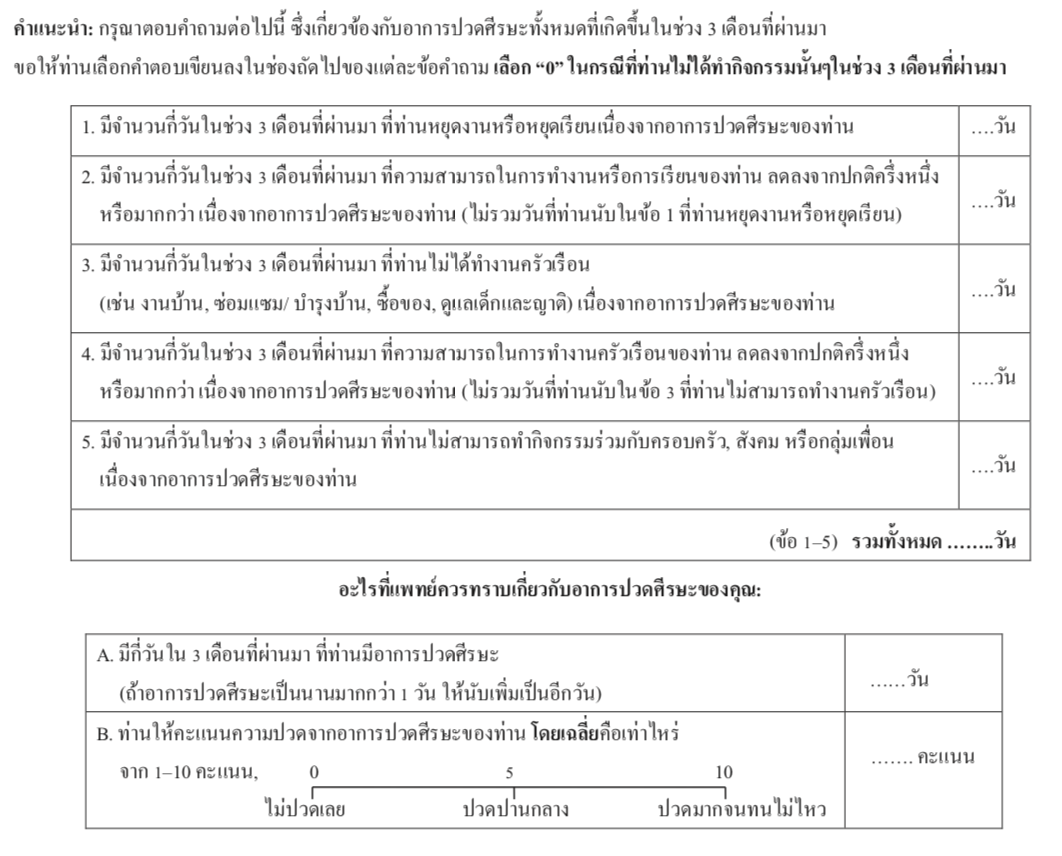


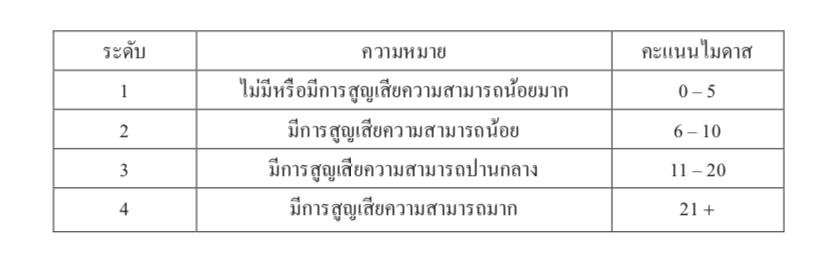


**Reference**: Vongvoivanich K, Youngprawat T, Jindawong N, et al. Test-Restest Reliablitiy of the Thai Migraine Disability Assessment (Thai-MIDAS) Questionanire in Thai Migraine Patients. The Bangkok Medical Journal. 2018;14 (1): 10-15.
